# Consumers’ Willingness to Pay for eHealth and Its Influencing Factors: Systematic Review and Meta-analysis

**DOI:** 10.2196/25959

**Published:** 2022-09-14

**Authors:** Zhenzhen Xie, Jiayin Chen, Calvin Kalun Or

**Affiliations:** 1 Department of Industrial and Manufacturing Systems Engineering The University of Hong Kong Hong Kong China (Hong Kong)

**Keywords:** systematic review, meta-analysis, willingness to pay, eHealth, contingent valuation, discrete choice experiment, mobile phone

## Abstract

**Background:**

Despite the great potential of eHealth, substantial costs are involved in its implementation, and it is essential to know whether these costs can be justified by its benefits. Such needs have led to an increased interest in measuring the benefits of eHealth, especially using the willingness to pay (WTP) metric as an accurate proxy for consumers’ perceived benefits of eHealth. This offered us an opportunity to systematically review and synthesize evidence from the literature to better understand WTP for eHealth and its influencing factors.

**Objective:**

This study aimed to provide a systematic review of WTP for eHealth and its influencing factors.

**Methods:**

This study was performed and reported as per the Cochrane Collaboration and PRISMA (Preferred Reporting Items for Systematic Reviews and Meta-Analyses) guidelines. PubMed, CINAHL Plus, Cochrane Library, EconLit, and PsycINFO databases were searched from their inception to April 19, 2022. We conducted random-effects meta-analyses to calculate WTP values for eHealth (at 2021 US dollar rates) and meta-regression analyses to examine the factors affecting WTP.

**Results:**

A total of 30 articles representing 35 studies were included in the review. We found that WTP for eHealth varied across studies; when expressed as a 1-time payment, it ranged from US $0.88 to US $191.84, and when expressed as a monthly payment, it ranged from US $5.25 to US $45.64. Meta-regression analyses showed that WTP for eHealth was negatively associated with the percentages of women (β=−.76; *P*<.001) and positively associated with the percentages of college-educated respondents (β=.63; *P*<.001) and a country’s gross domestic product per capita (multiples of US $1000; β=.03; *P*<.001). Compared with eHealth provided through websites, people reported a lower WTP for eHealth provided through asynchronous communication (β=−1.43; *P*<.001) and a higher WTP for eHealth provided through medical devices (β=.66; *P*<.001), health apps (β=.25; *P*=.01), and synchronous communication (β=.58; *P*<.001). As for the methods used to measure WTP, single-bounded dichotomous choice (β=2.13; *P*<.001), double-bounded dichotomous choice (β=2.20; *P*<.001), and payment scale (β=1.11; *P*<.001) were shown to obtain higher WTP values than the open-ended format. Compared with ex ante evaluations, ex post evaluations were shown to obtain lower WTP values (β=−.37; *P*<.001).

**Conclusions:**

WTP for eHealth varied significantly depending on the study population, modality used to provide eHealth, and methods used to measure it. WTP for eHealth was lower among certain population segments, suggesting that these segments may be at a disadvantage in terms of accessing and benefiting from eHealth. We also identified the modalities of eHealth that were highly valued by consumers and offered suggestions for the design of eHealth interventions. In addition, we found that different methods of measuring WTP led to significantly different WTP estimates, highlighting the need to undertake further methodological explorations of approaches to elicit WTP values.

## Introduction

Advances in broadband technology and the Internet of Things have enabled the broad implementation of eHealth—the provision or acquisition of health information or services through electronic processes [1-7]. In recent years, a broad spectrum of eHealth interventions using various modalities has been developed and examined in health care research. Examples include websites, diagnostic and monitoring devices, smartphone apps, virtual reality systems, telephone and video calls, and electronic messages that provide health information or services [8-10]. Researchers have implemented these eHealth interventions into a range of health care activities, including teleconsultation [11,12], remote patient monitoring [13], self-management of diseases [14-16], disease rehabilitation [17], and disease prevention [18]. Promising results have emerged from these studies, which showed that eHealth interventions could facilitate the delivery of health care and improve patient outcomes [9-17]. It has also been shown that eHealth enables consumers to easily obtain information about health issues for decision-making, which could lead to more effective care, patient empowerment, and time savings [8,18-22].

Although eHealth is considered a promising complement to conventional health care systems, there are significant costs involved in its implementation arising from the purchase, development, and maintenance of hardware and software [23]. Therefore, when deciding to implement eHealth for personal use or public health, decision-makers need solid evidence that the costs of eHealth can be justified by its benefits [24]. This requires the quantification and measurement of the benefits of eHealth, which can then be aggregated with the costs of eHealth to understand its cost-effectiveness [25].

To measure the benefits of eHealth, willingness to pay (WTP) is a commonly used metric [26,27]. Welfare economics defines WTP as the maximum amount of money an individual is willing to pay for 1 unit of a good or service; it is an accurate proxy for the welfare (benefits) derived from that good or service [28-30]. A major advantage of the WTP approach is that it summarizes the benefits in monetary terms, making it comparable with the costs for use in cost-benefit analyses [26,31]. Another advantage is that WTP illustrates the perceived benefits from the perspective of consumers, which can be further analyzed to represent consumer preferences [32,33]. Therefore, the WTP approach is suitable for measuring the benefits of eHealth, as it can generate findings for the effective implementation of eHealth and provide insights into designing better eHealth technology and services.

Many studies [34-36] have examined consumers’ WTP for eHealth using either of the 2 mainstream methods. The first is contingent valuation, a survey-based method in which people are asked to indicate the maximum price they are willing to pay for eHealth (eg, services) or associated eHealth technology. The second is the discrete choice experiment, sometimes referred to as conjoint analysis, which involves asking people to state their preference for hypothetical alternatives that describe eHealth or eHealth technology. Regardless of the methods used, these studies have provided insights into consumers’ perceived eHealth benefits and the factors affecting these perceptions. If we synthesize and analyze these studies, we can obtain practical implications for the design, development, and implementation of eHealth and suggestions for future research. Thus, we systematically reviewed previous studies on consumers’ WTP for eHealth and synthesized their findings through a meta-analysis to understand consumers’ WTP for eHealth and examine its influencing factors. To the best of our knowledge, this is the first study of its kind.

## Methods

### Overview

This review was performed and reported according to the Cochrane Collaboration [37] and PRISMA (Preferred Reporting Items for Systematic Reviews and Meta-Analyses) guidelines (Multimedia Appendix 1 provides the checklist) [38]. A total of 2 researchers (ZX and JC) independently screened the titles and abstracts of the articles identified in the literature search for eligibility, reviewed the full texts of potentially eligible articles for final inclusion in the review, extracted data from the final sample, critically appraised their methodological quality, and assessed the quality of the evidence. All disagreements between them were resolved through a consensus-based discussion.

### Search Strategy

We searched PubMed, CINAHL Plus, Cochrane Library, EconLit, and PsycINFO databases from their inception to April 19, 2022, to obtain a preliminary list of relevant studies. A search strategy was developed based on the following concepts combined using “AND”: WTP, money, and eHealth. For each concept, a set of keywords and their synonyms and variations were developed and combined in the search strategy using “OR.” The following search terms were developed: (“willingness to pay” *OR* “WTP” *OR* “valuation” *OR* “preference”) *AND* (“cost” *OR* “price” *OR* “expense” *OR* “money”) *AND* (“eHealth” *OR* “electronic health” *OR* “digital health” *OR* “mHealth” *OR* “mobile” *OR* “web” *OR* “Internet” *OR* “online” *OR* “tele*” *OR* “medical informatics” *OR* “medical information systems”). These search terms were used to search for titles and abstracts in all the selected databases, with no filters or limits placed on the search.

### Eligibility Criteria and Study Selection

We included all studies that (1) recruited participants who were consumers of eHealth, (2) measured and reported participants’ WTP for eHealth or eHealth technology, and (3) were published in a peer-reviewed English-language journal. Studies were excluded if they examined WTP from a public payer’s perspective (eg, WTP for public health programs through taxation) or a caregiver’s perspective (eg, parents’ WTP for their children). We also excluded reviews, case studies, poster presentations, and conference presentations but examined their references to identify additional relevant articles for inclusion. We also manually searched the reference lists of studies in the final sample for additional relevant articles.

### Data Extraction and Management

We extracted the following data from each study: country where the study was conducted, year in which it was conducted, sample size, sample characteristics, modality used to provide eHealth, details of the eHealth examined, WTP, method used to measure WTP, and WTP factors examined. Regarding the methods used to measure WTP, the extracted information included the formats of the questions posed to the study participants (eg, open-ended questions, dichotomous choice, and bidding games), whether the participants had used eHealth at the time of evaluation (ex post or ex ante), and how zero responses were dealt with (all zero responses excluded, all zero responses included, or protest zero responses excluded). We contacted the authors for clarification and verification of cases where relevant data were missing or incomplete.

### Critical Appraisal of Methodological Quality

The included studies were critically appraised for methodological quality using 17 criteria based on the Hoy risk of bias assessment tool [39] and a set of criteria specific for assessing WTP studies (Multimedia Appendix 2) [29,40].

### Data Analysis

#### Descriptive Statistics and Narrative Synthesis of the Studies in the Final Sample

Descriptive statistics were used to summarize the characteristics of the included studies. Narrative synthesis was used to synthesize the WTP findings for eHealth in the studies, for which the means, SDs, 95% CIs, medians, IQRs, and ranges were reported. All WTP values were calculated at 2021 US dollar rates to facilitate quantitative synthesis and comparison. First, the WTP values in other currencies were converted to US dollars based on the purchasing power parity (PPP) exchange rate of the year in which the study was conducted, and then they were converted to 2021 US dollar values using gross domestic product (GDP) deflators. The PPP exchange rate and GDP deflator data were obtained from the International Monetary Fund’s World Economic Outlook database [41]. For studies that did not report the year in which they were conducted, we used the year preceding the publication year of the articles for currency conversion.

#### Random-Effects Meta-analyses to Measure WTP

We performed random-effects meta-analyses to estimate the overall WTP value for eHealth and the WTP value for eHealth by different subgroups (ie, modalities used to provide eHealth and the region where the study was conducted) [42]. The WTP values were log-transformed to reduce skewness [43]. In the meta-analysis, the weight of each study was the inverse of the WTP variance. For studies that did not report variance (or SD), we obtained an estimate using (1) SE and sample size, (2) 95% CIs and sample size, (3) IQRs, or (4) range and sample size [37,44]. The *I*^2^ test was used to measure heterogeneity in the synthesized studies [45], and the Egger test was used to assess the possibility of publication bias [46].

#### Meta-regression Analyses to Examine the Factors Affecting WTP

Univariate meta-regressions were conducted to examine whether WTP for eHealth was influenced by explanatory variables, including gender, age, and education level of the study sample; per capita GDP of the country where the study was conducted and the year in which it was conducted; the modality used to provide eHealth (ie, websites, medical devices, health apps, asynchronous communication, and synchronous communication); the format of the WTP questions (ie, open-ended, single-bounded dichotomous choice, double-bounded dichotomous choice, or payment scale); whether the participants of the study had used eHealth at the time of evaluation (ex post vs ex ante); and whether zero responses were excluded from the analysis of WTP values. A mixed-effects log-linear regression model was used, where the payment horizon (1-time or monthly payment) was modeled as a random effect and the explanatory variable was modeled as a fixed effect. We also narratively synthesized the WTP factors for eHealth examined in the included studies. All statistical analyses were performed in R (version 4.0.2, R Foundation for Statistical Computing) software using the *metafor* package.

### Assessment of the Quality of Evidence

The quality of evidence of the meta-analysis results was assessed using the Grading of Recommendations, Assessment, Development, and Evaluation (GRADE) framework [47]. We adopted the framework for rating the relative importance of outcomes (eg, values, preferences, and outcome importance) [48,49], which was more suitable for rating cross-sectional WTP surveys and discrete choice experiments than previous GRADE guidelines that focused on the effects of interventions. For each WTP outcome, the quality of evidence started from “high” and was downgraded by 1 level for every serious issue identified in the domains of risk of bias, inconsistency, indirectness, imprecision, and publication bias. The risk of bias domain was assessed by inspecting the potential bias in participant selection, measurement instruments, data collection, and data analysis. The inconsistency domain was assessed using *I*^2^ values, and the GRADE quality was downgraded when *I*^2^≥50%. The indirectness domain was assessed using the indirectness of the population, outcomes, options, and methodologies used to elicit the values of the outcomes. The imprecision domain was assessed using the width of the CIs of the estimates and sample size. The publication bias domain was assessed using the Egger test, and GRADE quality was downgraded for statistically significant findings (*P*<.05) on this test.

## Results

### Literature Search and Selection Process

**Figure 1:**
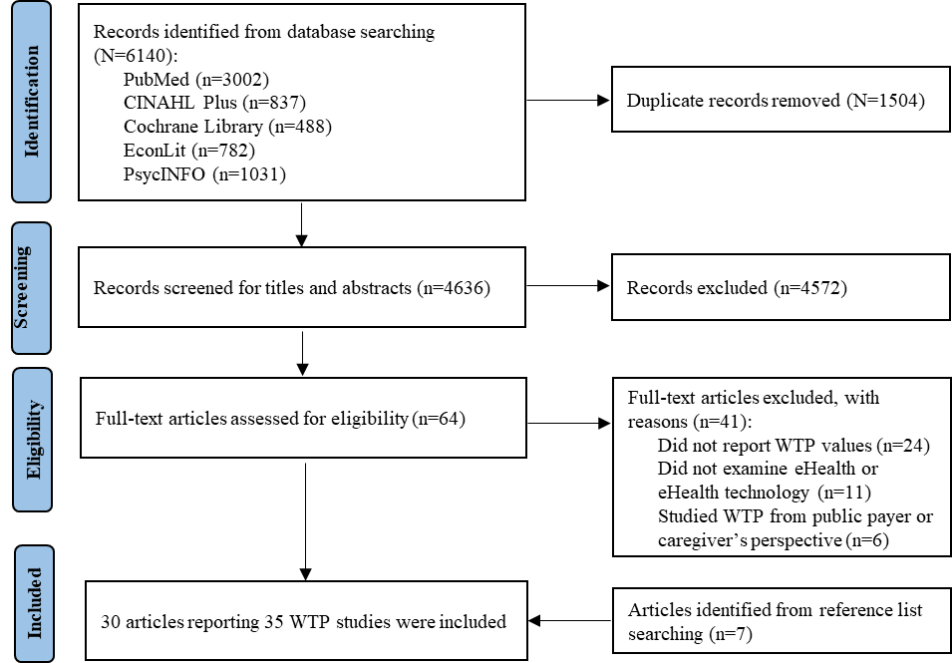
PRISMA (Preferred Reporting Items for Systematic Reviews and Meta-Analyses) flow diagram of the study selection process. WTP: willingness to pay.

Figure 1 shows the literature search and selection process. The search yielded 6140 articles, of which 30 (0.49%) articles representing 35 WTP studies were identified as eligible and included in the final review.

### Study Characteristics

The characteristics of the studies included in this review are presented in Table 1. Appraisals of the methodological quality are presented in Multimedia Appendix 2.

**Table 1:** Summary of the characteristics of the final studies (N=35).

Characteristics			Values, n (%)
**Study location**			
	Africa		2 (6)
	Asia		8 (23)
	Europe		13 (37)
	North America		9 (26)
	Oceania		3 (9)
**Year of publication**			
	2003-2010		8 (23)
	2011-2015		8 (23)
	2016-2021		19 (54)
**Modality used to provide eHealth**			
	Websites		5 (14)
	Medical devices		8 (23)
	Health apps		5 (14)
	Asynchronous communication (eg, SMS text messaging or email)		8 (23)
	Synchronous communication (eg, telephone call or video call)		7 (20)
	Not specified		2 (6)
**Method used to measure willingness to pay**			
	**Contingent valuation**		26 (74)
		Open-ended questions	13 (37)
		Single-bounded dichotomous choice questions	1 (3)
		Double-bounded dichotomous choice questions	4 (11)
		Payment scale questions	2 (6)
		Bidding games	2 (6)
		Single-bounded dichotomous choice+payment scale questions	1 (3)
		Not reported	3 (9)
	Discrete choice experiment		9 (26)

### WTP for eHealth: Narrative Synthesis

Table 2-Table 4 present the details of 74% (26/35) of studies that used contingent valuations and 26% (9/35) of studies that used discrete choice experiments.

**Table 2:** Details of the 26 contingent valuation studies included in the final sample.

Study			Country (year of study)		Population and sample size (N)		Age (years)		Women (%)		eHealth details		Measurement of WTP^a^ (format, ex ante or ex post, and zeros)		WTP (PPP^b^, and 2021 US dollar value)
**Contingent valuation studies that reported** **WTP** **as a 1-time payment (n=17)**															
	Adedokun et al [50]	Nigeria (2011)		Patients at a family medicine unit (389)		Mean 42.1		54		An SMS text messaging–based appointment scheduling service: patients sent an SMS text message to book a clinic appointment and received a confirmation SMS text message and another SMS text message reminding them of the appointment		Open-ended; ex ante; all zeros excluded		Mean 2.81 (SD 3.88), range 0.06-38.26	
	Belkora et al [51]	United States (2007-2010)		Patients with breast cancer (34)		Mean 59		100		A telephone consultation planning service: before a clinical visit, a community health worker called the patient to check if they had any medical questions and then sent the list of questions to the patient’s physician		Double-bounded dichotomous choice; ex post; all zeros included		Mean 191.84 (SD 242.91)	
	Bergmo and Wangberg (1) [52]	Norway (2003)		Patients at a primary clinic (52)		Mean 38		70		An internet-based messaging system that enabled patients to communicate with their health care providers by sending messages using a web browser		Open-ended; ex ante; protest zeros excluded		Mean 10.94 (95% CI 8.91-13.17); median 10.14 (IQR 5.07-20.26)	
	Bergmo and Wangberg (2) [52]	Norway (2003)		Patients at a primary clinic (38)		Mean 37		61		Same as Bergmo and Wangberg (1) [52]		Open-ended; ex post; protest zeros excluded		Mean 7.30 (95% CI 5.47-8.91); median 7.09 (IQR 2.03-10.14)	
	Brandling-Bennet et al [53]	Cambodia (2003)		Patients at a clinic (49)		Mean 39		61		A telemedicine service: local nurses recorded the medical history and conducted physical examinations of patients and sent this information to physicians at a remote place via email; the physicians would then reply with the treatment or referral decisions; the local nurses would execute the recommendations		Not reported; ex post; all zeros excluded		Median 0.90, range 0-72.53	
	Fawsitt et al (1) [54]	Ireland (2015)		Women in antenatal clinics (20)		Mean and median not reported		100		A mobile app that provided information about cesarean section and surgical site infections: users recorded symptoms, temperature, heart rate, and pain level based on which the app would provide health advice (eg, check body temperature or contact a general practitioner)		Open-ended; ex ante; all zeros included		Mean 30.96 (SD 58.28); median 13.98	
	Fawsitt et al (2) [54]	Ireland (2015)		Women in antenatal clinics (116)		Mean and median not reported		100		A mobile app that provided information about cesarean section and surgical site infections: users recorded symptoms, temperature, heart rate, and pain level, which would be checked daily by a midwife in the maternity hospital who would provide health advice to the user		Open-ended; ex ante; all zeros included		Mean 36.38 (SD 51.46); median 13.98	
	Fawsitt et al (3) [54]	Ireland (2015)		Women in antenatal clinics (44)		Mean and median not reported		100		A telephone call–based helpline service: users called a midwife in the maternity hospital, who would provide health advice and instructions		Open-ended; ex ante; all zeros included		Mean 32.76 (SD 47.73); median 13.98	
	Kaga et al [55]	Japan (2016)		General population (305)		Mean and median not reported		37		An internet-based telecare service for older adults, which connected the television at users’ homes to the internet: health care information was displayed on the television; if the television was not used for 3 days, a telephone call would be made to the user, and if they did not answer the call, neighborhood associations and civil servant committees would visit them to ensure that they were fine		Double-bounded dichotomous choice; ex ante; all zeros included		Mean 8.58; median 4.57	
	Ngan et al [56]	Vietnam (2017)		Smokers who intended to quit (433)		Mean 33		0.8		An SMS text messaging–based smoking cessation service: SMS text messages with relevant health information, suggestions for controlling and preventing cravings, and encouragement were sent to users 2 to 4 times a day for 6 weeks		Single-bounded dichotomous choice; ex ante; all zeros included		Mean 59.99 (95% CI 46.92-73.07)	
	Raghu et al (1) [57]	United States (2013-2014)		Patients waiting for general consultation (214)		Mean and median not reported		Not reported		A teledermoscopy service: a clinician at a health center used a smartphone (with a Canfield Dermscopefield) to capture images of skin lesions and send them to a dermatologist, who then wrote a medical note and sent it to the clinician		Double-bounded dichotomous choice; ex ante; all zeros included		Mean 63.12 (SD 44.66); median 55.77	
	Raghu et al (2) [57]	United States (2013-2014)		Patients with skin lesions (41)		Mean and median not reported		Not reported		Same as Raghu et al (1) [57]		Double-bounded dichotomous choice; ex ante; all zeros included		Mean 59.81 (SD 30.33); median 54.83	
	Ramchandran et al [58]	United States (2017)		Patients with diabetes (23)		Mean 56		52		A teleophthalmology service: a technician or nurse used a nonmydriatic fundus camera to take photos of the patient’s eye and send them to an ophthalmologist, who then replied with a diagnosis and recommended follow-up care		Payment scale; ex ante; all zeros included		Mean 29.96 (SD 8.53)	
	Rochat et al [59]	Switzerland (2014)		People visiting a travel clinic (162)		Mean and median not reported		53		A telemedicine service for travelers providing pretravel information; medical advice for upcoming trips; and health advice when the traveler was abroad through telephone calls, video calls, or emails		Not reported; ex ante; all zeros excluded		Median 57.10 (IQR 34.26-57.10)	
	Ruby et al [60]	United States (2008)		Adolescents with persistent subthreshold depression (34)		Mean 17		57		An internet-based depression prevention intervention for adolescents: 14 modules for depression prevention were provided through a website		Not reported; ex post; all zeros included		Median 50.15 (IQR 19.59-62.68); range 0-626.84	
	Shariful Islam et al [61]	Bangladesh (2013-2014)		Patients with type 2 diabetes (352)		Mean 50		56		An SMS text message–based health service for patients with type 2 diabetes, which provided medication reminders and relevant health information (eg, diabetes complications and recommended diet and physical activities) through SMS text messages		Open-ended; ex ante; all zeros included		Median 0.88 (IQR 1.99)	
	Stahl et al [62]	United States (2007-2008)		Patients visiting a primary care physician (101)		Mean 46		60		An internet-based primary care service: a primary care physician took the patient’s medical history, conducted a visual inspection, decided on treatment, and arranged follow-up care through videoconferencing		Payment scale; ex post; all zeros included		Mean 25.71 (SD 15.88)^c^	
**Contingent valuation studies that reported** **WTP** **as monthly payments: WTP per month (n=9)**															
	Cocosila et al [63]	Canada (2006-2007)		General population (51)		Median 21		57		An SMS text message–based health reminder service: users received SMS text messages reminding them to take vitamin C pills		Open-ended; ex post; no zero responses		Median 5.25; range 0.52-31.47	
	Contreras-Somoza et al [64]	Spain, Serbia, Netherlands, France, Israel, Italy, or Slovenia (not reported)		Patients aged >60 years with mild cognitive impairment (30)		Mean 73.3		60		An internet-based information and communication technology platform (ehcoBUTLER system) for older people: the platform hosted several social and health apps to support the daily activities of older people and improve their health, quality of life, and independence		Not reported; ex ante; all zeros excluded		Median 14.64	
	Jacobs et al [65]	Belgium (2009)		General population (135)		Mean 41		34		A cardiovascular disease prevention program with internet-based components: the program comprised cardiovascular risk assessment, communication, follow-up care, a website providing health information on cardiovascular disease, advice on physical activity and diet, guidelines for behavioral changes, and individual coaching by a health psychologist		Single-bounded dichotomous choice+payment scale; ex post; all zeros included		Mean 13.41 (SD 14.42); median 5.64	
	Rasche et al [66]	Germany (2017)		General population (96)		Mean 63.8		51		A mobile app for fall prevention: the app had features such as detecting the risk of falling, recommendations for reducing this risk, storing other health-related data, and providing advice on how to prevent and respond to a fall		Open-ended; ex ante; all zeros included		Median 7.41 (IQR 14.83); range 0-118.61	
	Somers et al (1) [34]	United Kingdom (2015)		General population (1697)		Mean 47		51		A mobile app for improving well-being outcomes: the app had features such as calling and messaging friends or families or local health care providers, setting health goals, tracking health status, sharing health data, and receiving information about the local community		Open-ended; ex ante; all zeros included		Mean 24.31; median 7.46; range 0-1344.36	
	Somers et al (2) [34]	United Kingdom (2015)		General population (305)		Mean 48		72		Same as Somers et al (1) [34].		Open-ended; ex ante; ell zeros included		Mean 20.13; median 7.46; range 0-896.62	
	Tran et al [67]	Vietnam (2012)		Patients with HIV or AIDS (1016)		Mean 35.4		36		A mobile phone–based medication reminder service for patients with HIV: SMS text messages, telephone calls, or automated voice calls were used to remind patients to take their medication on time		Not reported; ex ante; all zeros included		Mean 8.42	
	Tsuji et al [68]	Japan (not reported)		General population (291)		Mean and median not reported		Not reported		A telehealth system for older people: health-related data such as blood pressure, oxygen saturation, heart rhythm, electrical activity, and heart rates were measured at the user’s home and sent to a remote clinic where nurses studied them and reported any unusual symptoms to the user and physicians; monthly health reports were created and sent to users		Bidding game; ex post; all zeros included		Mean 45.64	
	Tsuji et al [69]	Japan (not reported)		General population (145)		Mean 74		74		Same as Tsuji et al [68]		Bidding game; ex ante; all zeros included		Mean 29.68	

^a^WTP: willingness to pay.

^b^PPP: purchasing power parity.

^c^The WTP values were obtained by combining the WTP values for subgroups, as reported in the articles.

**Table 3:** Demographic and eHealth details of the 9 discrete choice experiment studies included in the final sample.

Study			Country (year of study)		Population		Sample size, N		Age (years)		Women (%)		eHealth details
**Discrete choice experiment studies that reported WTP^a^ as a 1-time payment (n=6)**													
	Buchanan et al [35]	United Kingdom (2018)		General population		734		Mean 47		51		Web-based consultation with a primary care physician	
	Park et al [70]	South Korea (2009-2010)		Patients in endocrinology and metabolism clinics		118		Mean 57		58		A telemedicine service for patients with diabetes	
	Snoswell et al [71]	Australia (not reported)		General population		113		Mean 40		74		A mobile teledermoscopy service for skin cancer screening: users used a dermoscopic smartphone attachment and app to take photos and send them to a dermatologist, along with relevant clinical information	
	Snoswell et al [36]	Australia (2019)		Patients who had a video consultation in the previous year		62		Mean and median not reported		62.9		Web-based consultation with a specialist physician through videoconferencing	
	Spinks et al [72]	Australia (not reported)		People aged 50 to 64 years at high risk of melanoma		35		Mean and median not reported		54		A teledermoscopy service for skin cancer screening: using a dermatoscope to take photos which were sent to a dermatologist for diagnosis	
	van der Pol and McKenzie [73]	United Kingdom (not reported)		General population		90		Mean and median not reported		62		A telemedicine service for ear, nose, and throat examination: patients sent endoscopic images to and videoconferenced with a specialist	
**Discrete choice experiment studies that reported** **WTP** **as monthly payments (n=3)**													
	Ahn et al [74]	South Korea (2011)		General population		400		Mean 44		51		A telemedicine service system that measured vital signs of users and transmitted patient data to care providers	
	Chang et al [75]	United States (2009-2010)		General population		6271		Mean and median not reported		52		A web-based health service that provided remote diagnosis, treatment, monitoring, and consultation	
	Deal et al [76]	Canada (not reported)		Patients with cardiovascular disease		74		Mean 68.9		50		A web-based system that tracked and displayed patients’ details on 15 outcomes related to cardiovascular disease risk, the target value of these outcomes for better control of their condition, the last time the outcome was checked, and brief advice for patients and clinicians	

^a^WTP: willingness to pay.

**Table 4:** WTP^a^ details of the 9 discrete choice experiment studies included in the final sample.

Study, attribute (reference level), and desired level or levels of the attribute					Marginal WTP (PPP^b^, 2021 US dollar value)
**Discrete choice experiment studies that reported WTP as a 1-time payment (n=6)**					
	**Buchanan et al [35]**				
		**How similar was your consultation to a traditional “face-to-face” appointment (the same)**			
			Video consultation	–7.02	
			Symptoms submitted via an electronic form	–15.40	
		**How long did you have to wait for a consultation**			
			Reduced by 1 hour	0.22	
		**Reputation of the GP^c^ (2 stars)**			
			5 stars	13.65	
		**Collecting antibiotics (taking a paper prescription to a pharmacy located in the same building as the local medical center)**			
			Prescription emailed to a pharmacy in another building as the local medical center	–11.38	
		**Form of consultation (at local medical centers)**			
			Via the internet (–10.83)	–17.09	
	**Park et al [70]**				
		**Service platform (the internet)**			
			Mobile phone	22.72	
		**Service providers (small- and medium-sized hospitals and clinics)**			
			Large general hospitals	21.64	
		**Service scope (glucose management only)**			
			Comprehensive diabetes care	24.23	
		**Personalization of consultation (absent)**			
			Present	11.87	
		**24-hour service accessibility (absent)**			
			Present	10.27	
		**Reply time (within 3 days)**			
			Within 1 day	8.45	
		**Assurance of service (low assurance)**			
			High assurance	18.61	
		**System failure (system down 1%-5%)**			
			System down <1%	12.68	
		**Confidentiality (1%-5% confidentiality breaches)**			
			<1% confidentiality breaches	8.78	
	**Snoswell et al [71]**				
		**Method of screening (by a GP)**			
			Mobile teledermoscopy	0.88	
		**Time away from usual activities (>4 hours)**			
			3-4 hours	6.11	
			1-2 hours	53.75	
		**Chances of detecting a melanoma if one is present (65%–75%)**			
			85%-95%	54.37	
			≥95%	87.73	
		**Wait time for results (3 days)**			
			<4 hours	4.92	
		**Person reviewing the results (GP)**			
			Dermatologist	32.21	
		**Number of moles removed to find 1 melanoma (5)**			
			3	31.51	
	**Snoswell et al [36]**				
		**Type and mode of consultation (local in-person consultation with a generalist physician at a GP clinic or small hospital)**			
			In-person consultation with a specialist physician at a large metropolitan hospital	9.88	
			Videoconference with a specialist physician from a local GP clinic or small hospital	91.33	
			Videoconference with a specialist from home	33.53	
		**Time away from home, office, or usual activities, including travel (half a day)**			
			1 full day	–11.80	
			≥2 full days	–113.66	
		**Perceived benefit from the consultation (limited)**			
			Partial benefit	53.86	
			Benefit	111.28	
		**Consulted or not (attending a consultation)**			
			No consultation chosen	–175.14	
	**Spinks et al [72]**				
		WTP for teledermoscopy service, in addition to skin self-examination, GP screening, and clinic skin cancer screening		84.38	
	**van der Pol and McKenzie [73]**				
		**Type of clinic**			
			Telemedicine	773.31	
			Face-to-face	873.45	
		**Driving time (up to 30 minutes)**			
			30-60 minutes	–57.49	
			60-90 minutes	–74.18	
			2-4 hours	–155.77	
		**Wait time**			
			Each additional week	–27.82	
**Discrete choice experiment studies that reported WTP as a monthly payment: WTP per month (n=3)**					
	**Ahn et al [74]**				
		**Device type (smartphone)**			
			Smart home	138.29	
			Wearable device	632.49	
		**Service type (management of oxygen saturation level)**			
			Blood glucose	30.27	
			Blood pressure	–56.35	
		**Service tailoring (absent)**			
			Present	82.76	
		**Reply time (usual)**			
			1-hour reduction	3.57	
	**Chang et al [75]**				
		Per household		5.40^d^	
	**Deal et al [76]**				
		**Speed of adding new information to the system (2 weeks)**			
			1 week	5.70	
			48 hours	7.60	
			Overnight	2.85	
			1 hour	0	
		**Individual patient tracker values displayed (most recent values only)**			
			2 most recent	8.55	
			12-month history	13.31	
			5-year history	8.55	
			Complete history	–5.70	
		**Nurse coordinator tasks or duties (no nurse coordinator)**			
			Basic duties^e^	16.16	
			Basic duties and input data	20.91	
			Basic duties and information sessions	17.11	
			Basic duties, phone, and email	33.27	
			Basic duties and reminders	19.96	
		**Frequency of contacting nurse coordinator (no contact)**			
			1 day per month	6.65	
			2 days per month	10.45	
			1 day per week	5.70	
			2 days per week	10.91	
			5 days per week	1.90	
		**Number of visits to a physician per year (1)**			
			2	19.01	
			3	25.66	
			4	27.56	
			6	7.60	

^a^WTP: willingness to pay.

^b^PPP: purchasing power parity.

^c^GP: general practitioner.

^d^95% CI 3.79-7.02.

^e^Basic duties of the nurse coordinator: assist the physician in using the tracker, keep tracker information updated, and ensure action is taken to address uncontrolled cardiovascular disease risks.

### WTP for eHealth: Meta-analysis

Approximately 60% (21/35) of studies reported sufficient data for inclusion in the meta-analysis. Among the 21 studies, 16 (76%) reported that WTP was measured as a 1-time payment, whereas 5 (24%) reported that it was measured as monthly payments. Table 5 presents the mean WTP for eHealth obtained through the meta-analysis.

**Table 5:** Overall WTP^a^ for eHealth, WTP by the modality used to provide eHealth, and WTP by the region where the study was conducted (N=21).

Variables				Studies, n (%)		Sample size		WTP (PPP^b^, 2021 US dollars), mean (95% CI)		*I*^2^ (%)		Egger test					GRADE^c^ quality of evidence
												*z* score		*P* value			
**Studies that measured** **WTP** **as a 1-time payment (n=16)** **[50-62]**																	
	Overall WTP			16 (76)		2102		25.00 (12.79-48.87)		99.69		0.72		.47		Low	
	**Modality used to provide eHealth**																
		Websites	1 (5)		34		111.46 (84.55-146.92)		N/A^d^		N/A		N/A		Very low		
		Medical devices	3 (14)		278		48.34 (30.17-77.44)		97.86		0.10		.92		Low		
		Health apps	2 (10)		136		35.86 (28.05-45.85)		N/A		N/A		N/A		Low		
		Asynchronous communication (eg, SMS text messages and email)	6 (28)		1313		7.76 (2.39-25.21)		99.55		1.67		.10		Low		
		Synchronous communication (eg, telephone and video call)	4 (19)		341		52.59 (22.15-124.90)		99.23		0.74		.46		Very low		
	**Region**																
		North America	6 (28)		447		61.92 (33.94-112.97)		99.01		3.30		.001		Low		
		Europe	6 (28)		432		22.65 (12.05-42.60)		98.16		0.24		.81		Moderate		
		Asia	3 (14)		834		9.93 (0.84-117.06)		99.76		1.3		.19		Low		
		Africa	1 (5)		389		2.81 (2.45-3.22)		N/A		N/A		N/A		Low		
**Studies that measured** **WTP** **as monthly payments: WTP per month (n=5)** **[34,63,65,66]**																	
	Overall WTP			5 (24)		2284		18.53 (11.81-29.08)		94.71		0.24		.81		Moderate	
	**Modality used to provide eHealth**																
		Websites	1 (5)		135		13.41 (11.19-16.08)		N/A		N/A		N/A		Moderate		
		Health apps	3 (14)		2098		28.89 (21.71-38.44)		44.49		−1.73		.08		Moderate		
		Asynchronous communication	1 (5)		51		10.62 (8.89-12.68)		N/A		N/A		N/A		Low		
	**Region**																
		North America	1 (5)		51		10.62 (8.89-12.68)		N/A		N/A		N/A		Low		
		Europe	4 (19)		2233		21.81 (13.91-34.20)		91.27		−0.12		.91		Moderate		

^a^WTP: willingness to pay.

^b^PPP: purchasing power parity.

^c^GRADE: Grading of Recommendations, Assessment, Development, and Evaluation.

^d^N/A: not applicable (as <3 experiments were analyzed).

Among the 16 studies that measured WTP as a 1-time payment, the mean WTP was US $25.00 (95% CI 12.79-48.87). The highest mean WTP was for eHealth provided through websites (US $114.46, 95% CI 84.55-146.92), followed by synchronous communication (US $52.59, 95% CI 22.15-124.90), medical devices (US $48.34, 95% CI 30.17-77.44), health apps (US $35.86, 95% CI 28.05-45.85), and asynchronous communication (US $7.76, 95% CI 2.39-25.21). In terms of region, the WTP value was the highest in North America (US $61.92, 95% CI 33.94-112.97), followed by Europe (US $22.65, 95% CI 12.05-42.60), Asia (US $9.93, 95% CI 0.84-117.06), and Africa (US $2.81, 95% CI 2.45-3.22).

Among the 5 studies that measured WTP as monthly payments, the mean WTP was US $18.53 (95% CI 11.81-29.08) per month. The highest mean WTP per month was for eHealth provided through health apps (US $28.89, 95% CI 21.71-38.44), followed by websites (US $13.41, 95% CI 11.19-16.08), and asynchronous communication (US $10.62, 95% CI 8.89-12.68). In terms of region, the mean WTP per month was US $21.81 (95% CI 13.91-34.20) in Europe and US $10.62 (95% CI 8.89-12.68) in North America.

### Factors Affecting WTP for eHealth: Meta-regression and Narrative Synthesis

Table 6 presents the results of the univariate log-linear meta-regression analyses of WTP-related factors for eHealth. The results showed that higher percentages of women (β=−.76; *P*<.001) were associated with a lower mean WTP value for eHealth, and more people with a college education (β=.63; *P*<.001) were associated with a higher mean WTP value for eHealth. No significant evidence was found to support the association between age and WTP for eHealth (*P*=.57). A higher GDP per capita was found to be related to a higher WTP value for eHealth (β=.03; *P*<.001). Compared with eHealth provided through websites, the respondents had a lower WTP value for asynchronous communication (β=−1.43; *P*<.001) and a higher WTP value for medical devices (β=.66; *P*<.001) and synchronous communication (β=.58; *P*<.001). Studies eliciting WTP values using the single-bounded dichotomous choice format (β=2.13; *P*<.001), double-bounded dichotomous choice format (β=2.20; *P*<.001), payment scale format (β=1.11; *P*<.001), and unspecified formats (β=1.89; *P*<.001) had higher mean WTP values than those using open-ended formats. Ex post evaluations had lower WTP values (β=−.37; *P*<.001) than ex ante evaluations. However, there was no significant difference in WTP between studies that excluded protest zero responses or all zero responses and studies that included all zero responses in their analysis (*P*=.37).

Among the studies included in this review, 40% (14/35) examined WTP-related factors for eHealth, and their findings were narratively synthesized (Tables 7 and 8). The factors of interest included the characteristics of the eHealth technology or service and the study participants’ sociodemographic characteristics, health conditions, current health care services, psychosocial characteristics, familiarity with information technology, and attitudes.

**Table 6:** Univariate log-linear meta-regression analyses of WTP^a^-related factors for eHealth.

Explanatory variable			Outcome variable (mean WTP)		
			β (SE, 95% CI)		*P* value
Gender (women; %)			−.76 (0.14, −1.03 to −0.49)		<.001
Age (years)			.002 (0.003, −0.004 to 0.01)		.57
Education (completed college; %)			.63 (0.18, 0.29 to 0.98)		<.001
GDP^b^ per capita (US $)			.03 (0.001, 0.025 to 0.027)		<.001
**Modality used to provide eHealth**					
	Websites	—^c^		—	
	Medical devices	.66 (0.08, 0.49 to 0.82)		<.001	
	Health apps	.25 (0.1, 0.06 to 0.44)		.01	
	Asynchronous communication (eg, SMS text messages and email)	−1.43 (0.09, −1.60 to −1.27)		<.001	
	Synchronous communication (eg, telephone and video call)	.58 (0.08, 0.42 to 0.74)		<.001	
**WTP** **question format**					
	Open-ended	—		—	
	Single-bounded dichotomous choice	2.13 (0.12, 1.90 to 2.36)		<.001	
	Double-bounded dichotomous choice	2.20 (0.06, 2.09 to 2.31)		<.001	
	Payment scale	1.11 (0.05, 1.01 to 1.21)		<.001	
	Not reported	1.89 (0.05, 1.80 to 1.98)		<.001	
Ex post vs ex ante			−.37 (0.04, −0.45 to −0.28)		<.001
Protest zero or all zero responses excluded vs all zeros included			.02 (0.02, −0.02 to 0.05)		.37

^a^WTP: willingness to pay.

^b^GDP: gross domestic product.

^c^Not available because it was the reference level.

**Table 7:** WTP^a^-related factors for the examined eHealth in studies that reported WTP as a one-time payment.

Factors			Adedokun et al [50]		Bergmo and Wangberg [52]		Kaga et al [55]		Ngan et al [56]		Raghu et al (1) [57]		Raghu et al (2) [57]		Shariful Islam et al [61]		Stahl et al [62]
**Characteristics of the eHealth or eHealth technology**																	
	Favorable features	—^b^		—		—		—		—		—		—		Positive^c^	
	Technical quality	—		—		—		—		—		—		—		Not significant	
	Service convenience	—		—		—		—		Positive		Positive		—		—	
	Satisfaction with the service	—		—		—		—		Not significant		Not significant		—		—	
	Brand reputation	—		—		—		—		Positive		Positive		—		—	
**Sociodemographic characteristics**																	
	Gender (women)	Not significant		Not significant		Negative		—		—		—		Negative		Not significant	
	Age	Not significant		Positive		Not significant		Positive		—		—		—		Not significant	
	Education	Not significant		Not significant		—		Not significant		Not significant		Negative		Positive		—	
	Income	—		Not significant		Not significant		Positive		Not significant		Positive		Positive		—	
	Employment	Not significant		—		—		—		Not significant		Not significant		—		—	
	Occupation	Not significant		—		—		Not significant		—		—		—		—	
	Living alone	—		—		Not significant		—		—		—		—		—	
	Residential area	—		—		—		Not significant		—		—		—		—	
	International student	—		—		—		—		Negative		Not significant		—		—	
**Health conditions**																	
	Chronic conditions	—		Not significant		—		—		—		—		Not significant		—	
	Smoking status	—		—		—		Not significant		—		—		—		—	
	Attempts to quit smoking	—		—		—		Not significant		—		—		—		—	
**Current health care services**																	
	Number of visits to a physician	—		—		—		—		—		—		Not significant		—	
	Time taken and cost of travel to see a physician	—		—		—		—		—		—		—		Not significant	
**Psychosocial characteristics**																	
	Health anxiety	—		—		Not significant		—		—		—		—		—	
	Health consciousness	—		—		Positive		—		—		—		—		—	
	Having an acquaintance who lives alone	—		—		Not significant		—		—		—		—		—	
	Not having seen people for over a week	—		—		Positive		—		—		—		—		—	
**Experience with information technology**																	
	Having used eHealth	—		Negative		—		—		—		—		—		—	
	Internet use	—		Not significant		—		—		—		—		—		—	
**Attitudes**																	
	Willingness to use	—		—		Positive		—		—		—		—		—	

^a^WTP: willingness to pay.

^b^The factor was not examined in the study.

^c^The favorable feature examined in the study was to involve family and friends.

**Table 8:** WTP^a^-related factors for the examined eHealth in studies that reported WTP in monthly payment.

Factors			Cocosila et al [63]		Jacobs et al [65]		Rasche et al [66]		Somers et al (1) [34]		Somers et al (2) [34]		Tran et al [67]
**Characteristics of the eHealth or eHealth technology**													
	Favorable features	—^b^		—		Positive^c^		—		—		Positive^d^	
**Sociodemographic characteristics**													
	Gender (women)	Not significant		—		Not significant		Not significant		Negative		—	
	Age	Negative		—		Not significant		Negative		Negative		—	
	Education	—		—		Not significant		—		—		Positive	
	Income	—		—		—		Positive		Not significant		—	
	Health literacy	—		—		Not significant		—		—		—	
**Health conditions**													
	Perceived health status	—		—		—		Positive		Positive		—	
	Chronic conditions	—		—		Not significant		Not significant		Not significant		—	
	Health risk	—		—		Not significant		—		—		—	
	Taking regular medication	—		—		—		Not significant		Not significant		Not significant	
	Dosage of medication	—		Positive		—		—		—		—	
**Current health care services**													
	Level of the health system	—		—		—		—		—		Negative	
**Psychosocial characteristics**													
	Perceived autonomy support	—		Positive		—		—		—		—	
**Experience with information technology**													
	Having used eHealth	—		—		—		—		—		—	
	Internet use	—		—		—		Positive and negative^e^		Not significant		—	
	SMS text messaging use	Not significant		—		—		—		—		—	
	Computer use	—		—		—		Negative^f^		Not significant		—	
	Smartphone use	—		—		—		Not significant		Not significant		—	
	Times without a mobile phone	—		—		—		—		—		Positive	
	Mobile app use	—		—		—		Not significant		Not significant		—	
	Amount spent on the phone, the internet, and additional features	—		—		—		Positive		Positive		Positive	
	Amount spent on health apps	—		—		—		Positive		Positive		—	
**Attitudes**													
	Attitude toward intervention	—		—		Not significant		—		—		—	
	Ready for technology innovation	—		—		Not significant		—		—		—	
	Willingness to use	—		—		—		—		—		Positive	

^a^WTP: willingness to pay.

^b^The factor was not examined in the study.

^c^Favorable features examined in the study included decisions regarding treatment, description of physical exercise to reduce the risk of falls, continuous workout programs, and making new social contacts.

^d^Favorable features examined in the study included direct counseling with physicians and booking check-ups.

^e^Individuals who had access to the internet at home but never used it showed higher WTP than those who did not have internet access at home; individuals who had access to the internet at home and used it regularly showed lower WTP than those who did not have internet access at home.

^f^Individuals who owned a computer but rarely used it showed a lower WTP than those who did not own a computer.

## Discussion

### Principal Findings

To the best of our knowledge, this study is the first systematic review and meta-analysis of WTP for eHealth and meta-regression analysis of WTP-related factors for eHealth. We summarized and analyzed the findings of relevant scientific papers and found that the WTP value reported in each study varied significantly depending on the study population, modality used to provide eHealth, and methods used to measure WTP.

WTP for eHealth was higher in North America and Europe than in Asia and Africa, which is in line with the positive association between GDP per capita and WTP found in our meta-regression analysis. These findings suggest that even after adjusting for PPP, the overall economic condition of a country is related to people’s WTP for eHealth. Furthermore, several studies have shown that individual or household income was positively associated with WTP for eHealth in their samples, suggesting that the economic condition of an individual also predicts their WTP for eHealth. A commonly cited reason for this finding is that economic conditions affect individuals’ ability to pay, which in turn affects their WTP [77]. Another reason may be that individuals with a higher income or those in more economically developed countries have better access to and are more familiar with eHealth and have a higher intention to use and pay for it [78].

The demographic characteristics related to WTP for eHealth were gender, age, and educational level. The meta-regression analysis showed that women were associated with lower WTP values, which is in line with the findings of some studies in which women were willing to pay less than men for eHealth [34,55,61]. A possible reason for this may be that men tend to be more concerned about their health because of the higher risks of life-threatening chronic diseases than women and are more willing to pay for tools to help manage their health conditions [79,80]. Another reason may be that men tend to have a more favorable attitude toward technology than women [81] and may be more likely to accept and favor eHealth. Regarding the association between age and WTP for eHealth, there were mixed results (ie, nonsignificant, significantly positive, and significantly negative associations) among the included studies. This suggests that the association may vary drastically, depending on the context of each study (eg, population, examined eHealth, clinical setting, and alternative health services). Educational level was also related to WTP for eHealth; studies with a higher percentage of college graduates reported higher WTP values than those with a lower percentage of college graduates. This could be explained by the fact that people with higher education levels had higher eHealth literacy levels [82], perceived fewer barriers to using eHealth, and were more willing to pay for eHealth.

People were more willing to pay for eHealth provided through a specific medical device (eg, dermatoscope, nonmydriatic fundus camera, or vital sign measurement system) than for eHealth provided through websites, probably because of the advantage of obtaining accurate measurements for better clinical diagnoses. The results also showed that people were more willing to pay for eHealth provided through synchronous communication (eg, telephone calls and videoconferencing) than for health-related websites that allow for little to no interaction between users and health care providers, probably because synchronous communication enables real-time communication between users and their health care providers. Asynchronous eHealth also enables communication with health care providers through store-and-forward methods, such as SMS text messaging or email. However, the mean WTP for asynchronous eHealth was much lower than that for synchronous eHealth, probably because the timeliness of communication cannot be guaranteed through asynchronous eHealth, and the amount of health information delivered through SMS text messages or emails is limited.

The methods used to measure WTP also influenced the WTP values. Our meta-regression analysis showed that posing open-ended questions to participants resulted in lower WTP values than any other contingent valuation method. The reason may be that open-ended questions yield more 0 responses [83,84]; alternatively, answering “yes” and anchoring effects can occur when the dichotomous choice or payment scale approach is used [85]. The meta-regression analysis also revealed that ex post evaluations led to lower WTP values than ex ante evaluations, probably because individuals who had not used eHealth tended to have higher expectations and value it more. Another explanation may be that some eHealth interventions were less user-friendly [52] or failed to meet user needs in practice [86].

### Implications for Practice, Policy, and Future Research

The results of this review reflect the value of eHealth from the perspective of users, who are important sources of practical implications for the development and implementation of eHealth [87,88]. Our results showed that users place a high value on an eHealth technology that offers accurate diagnoses of health problems, has interactive features, and facilitates real-time communication with health professionals [89-91]. They also favor eHealth technology that enables shared decision-making, physical exercise training, socializing, and booking health examinations [62,66,67,92,93]. In addition, users find convenient and easy-to-use eHealth to be more attractive, suggesting that usability and technology acceptance should be taken into consideration when designing and implementing technology for eHealth, which is consistent with the literature [86,94-103].

Our results revealed the gender, education, and economic differences in the WTP for eHealth. Despite that eHealth has great potential to improve the accessibility of care by delivering health care and health information remotely and at a low cost; it might be more accessible to and create more benefits for individuals or populations that have more resources to use and are more capable of using eHealth [104]. It is a challenge for researchers, eHealth developers, and public health decision-makers to ensure that eHealth helps resolve health disparities instead of exacerbating them. We recommend identifying and removing barriers to eHealth access among disadvantaged populations [105] and keeping users’ needs and eHealth literacy levels in mind when developing eHealth interventions [106].

Our review showed that the most common approach to elicit WTP for eHealth was open-ended questions, as researchers do not have to provide cues for a reasonable WTP value, and it is easy to use. However, many participants may have never been asked these types of questions in real life and may have found it difficult to answer, leading to a low response rate and more zero responses, especially “protest zeros” [83,84]. In comparison, other formats that gave participants a starting value to consider, such as single- and double-bounded dichotomous choice models, payment scales, and bidding games, may have made it easier for them to answer the questions but could have led to anchoring bias by making the participants believe that the starting value was an appropriate value, which could have biased their responses toward that value [85]. Some studies used discrete choice experiments, in which each attribute of the good or service was valued separately instead of the full package. Discrete choice experiments generally have higher internal and external validity but require more time and effort for study design and data collection than contingent valuation studies [107]. The perfect approach for WTP evaluation remains debatable, and it seems that the approaches cannot substitute each other, which underscores the need to undertake further methodological comparisons between different approaches and explore other approaches to elicit WTP.

### Limitations

This study had some limitations that should be acknowledged. First, all the studies identified in this review were stated preference studies that used hypothetical questions to measure WTP values instead of observing actual purchases or choices made by the respondents (ie, revealed preferences). This inevitably led to a hypothetical bias, with participants reporting higher WTP values than what they would pay in real life [108-111]. The dearth of revealed preference studies in this field calls for further investigation into how much people are willing to pay for eHealth in real life and a comparison of WTP values elicited through stated preference and revealed preference methods. Second, articles written in languages other than English were excluded from this review, which may have led to language and publication bias. Third, there was great heterogeneity in the meta-analysis results, which limited the generalizability of the reported mean WTP values. Meta-analysis and meta-regression results should be interpreted with caution. Finally, we conducted a univariate meta-regression analysis as the rule of thumb is that the number of studies to be used in an analysis should be at least 10 times the number of explanatory variables in the regression [37]. Hence, this review did not use multivariate regression to control for all potential confounders and covariates when examining the associations between exploratory variables and WTP for eHealth.

### Conclusions

We found that WTP for eHealth varies greatly depending on the modality used to provide eHealth, study population, and methods used to measure WTP. We found that consumers favored and valued several eHealth modalities and features, which should be considered for adoption in future eHealth interventions. User-centered, convenient, and easy-to-use eHealth interventions should be developed, keeping in mind their usability and acceptance. Our results also showed that different population segments have significantly different WTP values for eHealth, which calls for further efforts to ensure the effective implementation of eHealth among disadvantaged populations and resolve health disparities. Thus far, there has been no consensus on the optimal approach to elicit WTP values, necessitating the exploration of other methods.

## Appendix

Multimedia Appendix 1 PRISMA (Preferred Reporting Items for Systematic Reviews and Meta-Analyses) checklist.

Multimedia Appendix 2 Critical appraisal of the methodological quality.

## Abbreviations

GDP: gross domestic product
GRADE: Grading of Recommendations, Assessment, Development, and Evaluation
PPP: purchasing power parity
PRISMA: Preferred Reporting Items for Systematic Reviews and Meta-Analyses
WTP: willingness to pay
