# Supplementary material for: Consumers’ Willingness to Pay for eHealth and Its Influencing Factors: Systematic Review and Meta-analysis
Source: J Med Internet Res. 2022 Sep 14;24(9):e25959. doi: 10.2196/25959 (PMC9520394; doi:10.2196/25959)
Supplement: Multimedia Appendix 2 [file jmir_v24i9e25959_app2.docx]

**Appendix**

**Table A2. Critical appraisal of the methodological quality.**

| Hoy's risk of bias assessment tool & other recommendations for WTP studies | | | Studies | | | | | | |
| --- | --- | --- | --- | --- | --- | --- | --- | --- | --- |
| Section | Item No. | Recommendation | Adedokun  et al., 2016 | Belkora  et al., 2012 | Bergmo and Wangberg, 2007 (1) | Bergmo and Wangberg, 2007 (2) | Brandling-Bennet et al., 2005 | Cocosila et al., 2008 | Contreras  -Somoza et al., 2020 |
| External validity | 1 | Was the study's target population a close representation of the national population in relation to relevant variables, e.g. age, sex, occupation? | No | No | No | No | No | No | No |
|  | 2 | Was the sampling frame a true or close representation of the target population? | No | No | No | No | No | No | No |
|  | 3 | Was some form of random selection used to select the sample, OR, was a census undertaken? | No | No | No | No | Yes | No | No |
|  | 4 | Was the likelihood of non-response bias minimal? | No | Yes | Yes | Yes | Yes | No | No |
| Internal validity | 5 | Were data collected directly from the subjects (as opposed to a proxy)? | Yes | Yes | Yes | Yes | Yes | Yes | Yes |
|  | 6 | Was an acceptable case definition used in the study? | Yes | Yes | Yes | Yes | Yes | Yes | Yes |
|  | 7 | Was the study instrument that measured the parameter of interest (e.g. prevalence of low back pain) shown to have reliability and validity (if necessary)? | Yes | Yes | Yes | Yes | No | Yes | No |
|  | 8 | Was the same mode of data collection used for all subjects? | Yes | Yes | Yes | Yes | Yes | Yes | Yes |
|  | 9 | Was the length of the shortest prevalence period for the parameter of interest appropriate? | N/A | N/A | N/A | N/A | N/A | N/A | N/A |
|  | 10 | Were the numerator(s) and denominator(s) for the parameter of interest appropriate? | N/A | N/A | N/A | N/A | N/A | N/A | N/A |
| Other criteria specific for WTP studies | 12 | Does a detailed description of goods or services in question offered to the respondents? | Yes | Yes | Yes | Yes | Yes | Yes | Yes |
|  | 13 | Does the information and attributes expressed in goods or services scenarios is obtained from user or key informant assessments (e.g. focus groups, Delphi panels, interviews etc)? | Yes | Yes | No | Yes | Yes | Yes | No |
|  | 14 | Was there a pilot study conducted to assess the survey tool/design? | No | No | No | No | No | No | No |
|  | 15 | Does the survey involve face to face interviews? | Yes | Yes | No | No | Yes | No | No |
|  | 16 | Was the usual payment amount (e.g. existing insurance premiums) used as a payment vehicle in study? | Yes | Yes | Yes | Yes | Yes | Yes | Yes |
|  | 17 | Is the time period of payment clear in the study tool? | Yes | No | Yes | Yes | Yes | Yes | Yes |

| Hoy's risk of bias assessment tool & other recommendations for WTP studies | | | Studies | | | | | | | | |
| --- | --- | --- | --- | --- | --- | --- | --- | --- | --- | --- | --- |
| Section | Item No. | Recommendation | Fawsitt et al., 2017 (1) | Fawsitt et al., 2017 (2) | Fawsitt et al., 2017 (3) | Jacobs et al., 2011 | Kaga et al, 2017 | Ngan et al., 2019 | Raghu et al, 2018 (1) | Raghu  et al., 2018  (2) | Ramchandron  et al, 2020 |
| External validity | 1 | Was the study's target population a close representation of the national population in relation to relevant variables, e.g. age, sex, occupation? | No | No | No | No | Yes | No | No | No | No |
|  | 2 | Was the sampling frame a true or close representation of the target population? | No | No | No | No | Yes | Yes | Yes | Yes | No |
|  | 3 | Was some form of random selection used to select the sample, OR, was a census undertaken? | No | No | No | No | No | No | No | No | No |
|  | 4 | Was the likelihood of non-response bias minimal? | Yes | Yes | Yes | Yes | Yes | No | No | No | No |
| Internal validity | 5 | Were data collected directly from the subjects (as opposed to a proxy)? | Yes | Yes | Yes | Yes | Yes | Yes | Yes | Yes | Yes |
|  | 6 | Was an acceptable case definition used in the study? | Yes | Yes | Yes | Yes | No | Yes | Yes | Yes | Yes |
|  | 7 | Was the study instrument that measured the parameter of interest (e.g. prevalence of low back pain) shown to have reliability and validity (if necessary)? | Yes | Yes | Yes | Yes | Yes | Yes | Yes | Yes | Yes |
|  | 8 | Was the same mode of data collection used for all subjects? | Yes | Yes | Yes | Yes | Yes | Yes | Yes | Yes | Yes |
|  | 9 | Was the length of the shortest prevalence period for the parameter of interest appropriate? | N/A | N/A | N/A | N/A | N/A | N/A | N/A | N/A | N/A |
|  | 10 | Were the numerator(s) and denominator(s) for the parameter of interest appropriate? | N/A | N/A | N/A | N/A | N/A | N/A | N/A | N/A | N/A |
| Other criteria specific for WTP studies | 12 | Does a detailed description of goods or services in question offered to the respondents? | Yes | Yes | Yes | Yes | No | Yes | Yes | Yes | Yes |
|  | 13 | Does the information and attributes expressed in goods or services scenarios is obtained from user or key informant assessments (e.g. focus groups, Delphi panels, interviews etc)? | No | No | No | Yes | No | No | No | No | No |
|  | 14 | Was there a pilot study conducted to assess the survey tool/design? | No | No | No | No | No | No | Yes | Yes | No |
|  | 15 | Does the survey involve face to face interviews? | No | No | No | No | No | Yes | No | No | Yes |
|  | 16 | Was the usual payment amount (e.g. existing insurance premiums) used as a payment vehicle in study? | Yes | Yes | Yes | Yes | Yes | Yes | Yes | Yes | Yes |
|  | 17 | Is the time period of payment clear in the study tool? | No | No | No | Yes | Yes | Yes | Yes | Yes | Yes |

| Hoy's risk of bias assessment tool & other recommendations for WTP studies | | | Studies | | | | | | | | | |
| --- | --- | --- | --- | --- | --- | --- | --- | --- | --- | --- | --- | --- |
| Section | Item No. | Recommendation | Rasche et al, 2018 | Rochat et al., 2018 | Ruby et al., 2013 | Shariful Islam et al, 2015 | Somers et al, 2019 (1) | Somers et al, 2019 (2) | Stahl et al., 2010 | Tran et al., 2012 | Tsuji et al, 2003 | Tsuji et al, 2006 |
| External validity | 1 | Was the study's target population a close representation of the national population in relation to relevant variables, e.g. age, sex, occupation? | No | No | No | No | Yes | No | No | No | No | No |
|  | 2 | Was the sampling frame a true or close representation of the target population? | No | No | No | No | Yes | Yes | No | Yes | No | No |
|  | 3 | Was some form of random selection used to select the sample, OR, was a census undertaken? | No | No | No | No | No | No | No | No | No | No |
|  | 4 | Was the likelihood of non-response bias minimal? | No | No | No | Yes | Yes | Yes | Yes | Yes | No | No |
| Internal validity | 5 | Were data collected directly from the subjects (as opposed to a proxy)? | Yes | Yes | Yes | Yes | Yes | Yes | Yes | Yes | Yes | Yes |
|  | 6 | Was an acceptable case definition used in the study? | Yes | Yes | Yes | No | Yes | Yes | Yes | Yes | Yes | Yes |
|  | 7 | Was the study instrument that measured the parameter of interest (e.g. prevalence of low back pain) shown to have reliability and validity (if necessary)? | Yes | No | No | Yes | Yes | Yes | Yes | No | Yes | No |
|  | 8 | Was the same mode of data collection used for all subjects? | Yes | Yes | Yes | Yes | Yes | Yes | Yes | Yes | Yes | Yes |
|  | 9 | Was the length of the shortest prevalence period for the parameter of interest appropriate? | N/A | N/A | N/A | N/A | N/A | N/A | N/A | N/A | N/A | N/A |
|  | 10 | Were the numerator(s) and denominator(s) for the parameter of interest appropriate? | N/A | N/A | N/A | N/A | N/A | N/A | N/A | N/A | N/A | N/A |
| Other criteria specific for WTP studies | 12 | Does a detailed description of goods or services in question offered to the respondents? | Yes | No | Yes | No | Yes | Yes | Yes | Yes | Yes | Yes |
|  | 13 | Does the information and attributes expressed in goods or services scenarios is obtained from user or key informant assessments (e.g. focus groups, Delphi panels, interviews etc)? | Yes | No | Yes | No | Yes | Yes | Yes | No | Yes | Yes |
|  | 14 | Was there a pilot study conducted to assess the survey tool/design? | Yes | No | No | No | Yes | Yes | No | No | No | No |
|  | 15 | Does the survey involve face to face interviews? | No | No | No | Yes | No | No | No | Yes | Yes | Yes |
|  | 16 | Was the usual payment amount (e.g. existing insurance premiums) used as a payment vehicle in study? | Yes | Yes | Yes | Yes | Yes | Yes | Yes | Yes | Yes | Yes |
|  | 17 | Is the time period of payment clear in the study tool? | Yes | Yes | No | Yes | Yes | Yes | Yes | Yes | Yes | Yes |

| Hoy's risk of bias assessment tool & other recommendations for WTP studies | | | Studies | | | | | | | | |
| --- | --- | --- | --- | --- | --- | --- | --- | --- | --- | --- | --- |
| Section | Item No. | Recommendation | Ahn et al., 2014 | Buchanan et al., 2021 | Chang et al., 2017 | Deal et al., 2014 | Park et al., 2011 | Snoswell et al., 2018 | Snoswell et al., 2021 | Spinks et al., 2016 | Van der Pol and McKenzie, 2010 |
| External validity | 1 | Was the study's target population a close representation of the national population in relation to relevant variables, e.g. age, sex, occupation? | Yes | No | Yes | No | No | No | No | No | Yes |
|  | 2 | Was the sampling frame a true or close representation of the target population? | Yes | No | Yes | No | No | No | Yes | No | No |
|  | 3 | Was some form of random selection used to select the sample, OR, was a census undertaken? | Yes | No | No | No | No | No | Yes | No | No |
|  | 4 | Was the likelihood of non-response bias minimal? | No | No | Yes | No | No | Yes | No | No | No |
| Internal validity | 5 | Were data collected directly from the subjects (as opposed to a proxy)? | Yes | Yes | Yes | Yes | Yes | Yes | Yes | Yes | Yes |
|  | 6 | Was an acceptable case definition used in the study? | Yes | Yes | Yes | Yes | Yes | Yes | Yes | Yes | Yes |
|  | 7 | Was the study instrument that measured the parameter of interest (e.g. prevalence of low back pain) shown to have reliability and validity (if necessary)? | Yes | Yes | Yes | Yes | Yes | Yes | Yes | Yes | Yes |
|  | 8 | Was the same mode of data collection used for all subjects? | Yes | Yes | Yes | Yes | Yes | Yes | Yes | No | Yes |
|  | 9 | Was the length of the shortest prevalence period for the parameter of interest appropriate? | N/A | N/A | N/A | N/A | N/A | N/A | N/A | N/A | N/A |
|  | 10 | Were the numerator(s) and denominator(s) for the parameter of interest appropriate? | N/A | N/A | N/A | N/A | N/A | N/A | N/A | N/A | N/A |
| Other criteria specific for WTP studies | 12 | Does a detailed description of goods or services in question offered to the respondents? | Yes | Yes | Yes | Yes | Yes | Yes | Yes | Yes | Yes |
|  | 13 | Does the information and attributes expressed in goods or services scenarios is obtained from user or key informant assessments (e.g. focus groups, Delphi panels, interviews etc)? | No | No | No | Yes | Yes | Yes | Yes | Yes | No |
|  | 14 | Was there a pilot study conducted to assess the survey tool/design? | No | No | No | No | Yes | Yes | Yes | No | No |
|  | 15 | Does the survey involve face to face interviews? | Yes | No | No | No | Yes | No | No | No | No |
|  | 16 | Was the usual payment amount (e.g. existing insurance premiums) used as a payment vehicle in study? | Yes | Yes | Yes | Yes | Yes | Yes | Yes | Yes | Yes |
|  | 17 | Is the time period of payment clear in the study tool? | Yes | Yes | Yes | Yes | Yes | Yes | Yes | Yes | Yes |
